# Rare glandular tumors of the nasopharynx: A retrospective cohort study of papillary adenocarcinoma and salivary gland–type carcinomas

**DOI:** 10.1097/MD.0000000000049047

**Published:** 2026-05-22

**Authors:** Dang Nguyen Van, Duc Nguyen Dinh, Nha Hoang Van, Toan Tran Trung

**Affiliations:** a Vietnam National Cancer Hospital – K Hospital, Hanoi, Vietnam; b Hanoi Medical University, Hanoi, Vietnam.

**Keywords:** nasopharyngeal carcinoma, papillary adenocarcinoma, salivary gland–type carcinomas, survival outcomes

## Abstract

Nasopharyngeal papillary adenocarcinomas (NPAC) and salivary gland–type nasopharyngeal carcinomas (SGNPCs) are rare tumors, accounting for approximately 0.38 to 0.48% of all nasopharyngeal malignancies. These tumors exhibit distinct clinical and pathological features and require different management approaches compared with conventional nasopharyngeal carcinoma. Given the limited literature, we conducted a retrospective study of 20 patients diagnosed with NPAC or SGNPC at our institution to summarize their clinical characteristics, treatment modalities, and outcomes. This retrospective study included 20 patients with pathologically confirmed NPAC or SGNPC treated at the Vietnam National Cancer Hospital from January 2019 to January 2026. A total of 20 patients were included, comprising 12 patients with NPAC and 8 patients with SGNPC. Tumors were mainly located at the roof or posterior margin of the nasopharynx, and most patients presented with early-stage disease (T1–T2) without cervical lymph node metastasis. All patients underwent endoscopic endonasal surgery, 6 patients were treated with surgery alone, while the remaining patients received postoperative radiotherapy using intensity-modulated radiation therapy. Postoperative complications were generally mild, including minor bleeding in one patient (5%) and dermatitis in all patients who received radiotherapy (100%). The median follow-up was 49.7 months (range, 4.5–84.0 months). The estimated 3-year and 5-year overall survival and disease-free survival rates were both 100% (95% confidence interval, 83.2–100%). Primary nasopharyngeal adenocarcinoma and salivary gland-type carcinoma are rare entities. In this cohort, the majority of patients presented at an early stage, and surgical intervention was the mainstay of treatment with favorable outcomes. However, these findings should be interpreted with caution given the small sample size and the surgically selected, early-stage nature of this cohort, and should be considered hypothesis-generating rather than definitive. Further prospective studies with larger patient populations and longer follow-up are needed to better define optimal treatment strategies and long-term outcomes for these rare tumors.

## 1. Introduction

Nasopharyngeal carcinoma is a malignant tumor arising from the epithelial lining of the nasopharyngeal mucosa. According to the current World Health Organization (WHO) classification, nasopharyngeal malignancies are categorized into carcinomas, primary nasopharyngeal papillary adenocarcinomas (NPACs), salivary gland-type nasopharyngeal cancers (SGNPCs) in addition to sarcomas/lymphomas and notochordal tumors.^[1]^ Nasopharyngeal carcinomas are the most common and are predominantly observed in Southern China and East Asia. For conventional nasopharyngeal carcinoma, established risk factors include Epstein–Barr virus (EBV) infection, occupational exposure to wood dust and formaldehyde, diets rich in smoked foods, as well as tobacco smoking and alcohol consumption. This tumor type is characterized by a high incidence of cervical lymph node metastasis and marked sensitivity to radiotherapy and chemotherapy, with radiotherapy constituting the cornerstone of treatment.

In contrast, nasopharyngeal papillary adenocarcinoma (NPAC) and salivary gland–type nasopharyngeal carcinoma (SGNPC) are rare entities, accounting for approximately 0.38 to 0.48% of all nasopharyngeal malignancies.^[2]^ Due to their rarity, most of these tumors have been reported primarily as isolated case reports or small case series, demonstrating clinicopathological features distinct from those of conventional nasopharyngeal carcinoma. Nasopharyngeal papillary adenocarcinoma was first described by Wenig et al.^[3]^ and represents a rare subtype of primary nasopharyngeal adenocarcinoma. These tumors are typically exophytic, exhibiting papillary, nodular, or polypoid growth patterns, and are generally associated with a favorable prognosis, with surgical resection serving as the primary treatment modality. Salivary gland–type nasopharyngeal cancers, however, are exceedingly rare and have therefore been historically difficult to investigate. Given that NPAC and salivary gland–type nasopharyngeal carcinomas arise from the same anatomical site, the American Joint Committee on Cancer (AJCC) TNM framework provides a standardized method for describing tumor extent and facilitating comparison across studies. However, its prognostic stratification may be limited in these rare histological subtypes due to their distinct biological behavior. Furthermore, the optimal treatment strategy, whether radiotherapy alone, surgery alone, or combined surgery with radiotherapy, remains controversial. Owing to the limited literature available, we conducted a retrospective study of 20 patients diagnosed with NPAC or SGNPC at our institution, aiming to summarize their clinical characteristics, treatment approaches, and outcomes.

## 2. Methods

### 2.1. Patients and study design

This retrospective study included 20 patients with pathologically confirmed nasopharyngeal papillary adenocarcinoma or salivary gland–type nasopharyngeal carcinoma. The patients were treated at the Vietnam National Cancer Hospital from January 2019 to January 2026. This study was reviewed and approved by the institutional review board and conducted in accordance with the Declaration of Helsinki.

We included all consecutive patients meeting the inclusion criteria between January 2019 and January 2026. Eligible patients were aged over 18 years, had histologically confirmed NPAC or SGNPC and were treated with curative intent by surgery with or without radiotherapy. Patients with an unclear staging, palliative treatment intent, lack of treatment, or incomplete follow-up data were excluded.

The tumor staging was performed according to the 8th edition of the AJCC staging system.

### 2.2. Treatment

The treatment methods consisted of surgery with or without postoperative radiotherapy.

Surgical treatment: All patients underwent complete tumor excision via an endoscopic endonasal approach. In principle, surgical margins were defined as the tumor invasion regions plus an additional 5 to 10 mm of peripheral mucosal margin and 2 to 3 mm of basal margin on the surface of the sphenoid bone and the clivus in the skull base. R2 resection is defined by macroscopic extension of the tumor to the resection margin with evidence of macroscopic residues. R1 resection is defined by microscopic extension of the tumor to the resection margin without evidence of macroscopic residues. Close margin was defined as a minimal distance of <2 mm between the tumor cells and the inked resection margin on histopathological examination. Indeterminate margin was defined as cases in which the resection margin was not assessed.

Radiotherapy: Indications for postoperative radiotherapy (PORT) included pT3–T4 primary tumors, pN2–N3 nodal disease, extranodal extension, positive/close/indeterminate margins, adverse pathological features (high-grade histology or adenoid cystic carcinoma), and perineural or vascular invasion. All patients were immobilized using a thermoplastic head-neck-shoulder mask to ensure accurate and reproducible positioning during radiotherapy. Both non-contrast CT scans for dose calculation and contrast-enhanced CT scans for target delineation were obtained, covering the region from the vertex to 2 cm below the sternoclavicular joints, with a slice thickness of 3 mm. The clinical target volume (CTV1) included macroscopic residual tumor (R2) or microscopically positive surgical margins (R1) with an isotropic margin of 5 mm. CTV2 comprised the preoperative gross tumor volume expanded by at least 10 mm, as well as CTV1 with an additional isotropic 5-mm margin, including all involved lymph nodes. Elective nodal irradiation in the N0 neck was not routinely performed due to the low risk of occult nodal metastasis and was reserved for selected high-risk cases (pT3–T4). The prescribed radiation dose ranged from 60 to 66 Gy delivered in 30 to 33 fractions, with a daily fraction size of 2 Gy. Dose constraints for organs at risk were applied in accordance with the QUANTEC guidelines for head and neck radiotherapy. Specifically, constraints were respected for critical structures including the spinal cord (maximum dose ≤ 45 Gy), brainstem (maximum dose ≤ 54 Gy), optic nerves and optic chiasm (maximum dose ≤ 54–55 Gy), parotid glands (mean dose ≤ 26 Gy when feasible), and temporal lobes (maximum dose ≤ 60 Gy).^[4]^

### 2.3. Study endpoint

Clinical and pathological data, including demographic characteristics, tumor location, histological subtype, immunohistochemical findings, treatment details, and outcomes, were extracted from medical records. This study assessed multiple survival endpoints, including overall survival (defined as the duration from date of diagnosis to death from any cause), disease-free survival (the time interval from date of diagnosis to either locoregional or distant metastatic recurrence, or death from any cause, whichever occurred first), locoregional recurrence-free survival (the period without confirmed locoregional relapse), and distant metastasis-free survival (the duration without evidence of distant metastatic spread).

### 2.4. Follow-up

Patients underwent regular clinical follow-up by an otolaryngologist and/or radiation oncologist. Follow-up visits were scheduled every 3 months during the first 2 years after treatment, every 6 months for the subsequent 3 years, and annually thereafter.

### 2.5. Statistical analysis

Categorical variables were compared between NPAC and SGNPC groups using Pearson chi-square test or Fisher exact test, as appropriate based on expected cell counts. Survival outcomes were estimated using the Kaplan–Meier method. However, due to the absence of events, formal survival comparisons between groups were not performed. Overall survival (OS) and disease-free survival (DFS) rates at clinically relevant timepoints (3-year and 5-year) were calculated, and exact binomial 95% confidence intervals (CIs) were reported.

## 3. Results

### 3.1. Demographic data and clinical features

From January 2019 to January 2026, a total of 20 patients with pathologically confirmed nasopharyngeal papillary adenocarcinoma or salivary gland–type nasopharyngeal carcinoma were included in this study. The median age was 50.5 years (range, 16–64), with a male-to-female ratio of 6:14. Most patients presented with early-stage disease, with 18 cases (90%) classified as T1–T2, and 2 cases (10%) classified as T3 at diagnosis; no cervical lymph node metastasis was observed. In the NPAC group, nasal bleeding was the most common presenting symptom (41.7%), followed by nasal obstruction (25%) and tinnitus (16.7%), while 16.7% of patients were asymptomatic. In the SGNPC group, nasal bleeding also predominated (37.5%), with nasal obstruction accounting for 25%, and headache and asymptomatic presentation each observed in 12.5% of cases. Macroscopically, a polypoid growth pattern was the most common, accounting for 60% of cases. In patients with nasopharyngeal papillary adenocarcinoma, tumors most commonly exhibited an exophytic or polypoid gross appearance, whereas a verrucous pattern predominated in salivary gland–type nasopharyngeal carcinoma. EBV-DNA status was available in 10 patients, all of whom tested negative; EBV testing was not performed in the remaining 10 patients. Histopathologically, nasopharyngeal papillary adenocarcinoma accounted for 12 cases (60%), while salivary gland–type nasopharyngeal carcinoma accounted for 8 cases (40%). Among salivary gland–type nasopharyngeal carcinomas, adenoid cystic carcinoma was the most common histological subtype (6 cases, 75%), followed by mucoepidermoid carcinoma (1 case, 12.5%) and clear cell carcinoma (1 case, 12.5%). Immunohistochemical staining for thyroglobulin was performed in 6 patients with nasopharyngeal papillary adenocarcinoma, and all cases were negative (Table 1).

**T-able 1:** General characteristics, tumor stage.

Characteristics	All patients			NPAC			SGNPC		
	n	%		n		%	n	%	
Gender									
Male	6	30		4		33.3	2	25	
Female	14	70		8		66.7	6	75	
Total	20	100		12		100	8	100	
Age	Median								
	50.5 (range, 16–64)			48.0 (range, 16–64)			57.5 (range, 46–64)		
Presenting symptoms									
Nasal bleeding	8		40	5	41.7		3		37.5
Nasal obstruction	5		25	3	25		2		25
Tinnitus	3		15	2	16.7		1		12.5
Headache	1		5	0	0		1		12.5
No symptom	3		15	2	16.7		1		12.5
Total	20		100	12	100		8		100
Location									
PMONP	1	5		0		0	1	12.5	
RON	13	65		12		100	1	12.5	
LWON	6	30		0		0	6	75	
Total	20	100		12		100	8	100	
T stage									
T1	9	45		8		66.7	1	12.5	
T2	9	45		4		33.3	5	62.5	
T3	2	10		0		0	2	25	
T4	0	0		0		0	0	0	
Total	20	100		12		100	8	100	
N stage									
N-Negative	20	100		12		100	8	100	
N-Positive	0	0		0		0	0	0	
Total	20	100		12		100	8	100	
Macroscopic appearance									
Exophytic	4	20		2		16.7	2	25	
Polypoid	12	60		10		83.3	2	25	
Infiltrating	0	0		0		0	0	0	
Verrucous	4	20		0		0	4	50	
Total	20	100		12		100	8	100	
EBV-DNA									
Positive	0	0		0		0	0	0	
Negative	10	50		4		33.3	6	75	
ND	10	50		8		66.7	2	25	
Total	20	100		12		100	8	100	
Pathological result									
NPAC	12	60		12		100	0	0	
Adenoid cystic carcinoma	6	30		0		0	6	75	
Mucoepidermoid carcinoma	1	5		0		0	1	12.5	
Clear cell carcinoma	1	5		0		0	1	12.5	
Total	20	100		12		100	8	100	
Thyroglobulin									
Positive	0	0		0		0	0	0	
Negative	6	30		6		50	0	0	
ND	14	70		6		50	8	100	
Total	20	100		12		100	8	100	

LWON = lateral wall of the nasopharynx, ND = not described, NPAC = primary nasopharyngeal papillary adenocarcinoma, PMONP = posterior margin of nasal septum, RON = roof of the nasopharynx.

### 3.2. Treatment and Follow-up

All patients underwent surgical treatment via an endoscopic endonasal approach. Among patients with salivary gland–type nasopharyngeal carcinoma, 2 patients were treated with surgery alone, while 6 patients underwent surgery followed by postoperative radiotherapy. No patients were treated with definitive radiotherapy alone. Among patients with NPAC (n = 12), margin status was R0 in 4 cases, R1 in 1 case, close in 4 cases, and indeterminate in 3 cases (Table 2). In the SGNPC group (n = 8), 3 patients had R0 margins, 1 had R1 margins, and 4 had indeterminate margins; no close margins were observed in this group (Table 2). All NPAC cases were low grade. Among salivary gland–type tumors, there was 1 high-grade adenoid cystic carcinoma (ACC) (grade 3), 5 low-grade ACCs, 1 intermediate-grade mucoepidermoid carcinoma, and 1 grade 1 clear cell carcinoma. Perineural invasion was observed in 2 ACC cases, while no perineural or lymphovascular invasion was identified in the other cases. The median follow-up for the entire cohort was 49.7 months (range, 4.5–84.0). It was 63.5 months (range, 4.5–84.0) for NPAC and 35.0 months (range, 5.4–78.9) for SGNPC. The estimated 3-year and 5-year OS and DFS rates were 100% (95% CI, 83.2–100%).

**Table 2:** Treatment and outcomes of the patients.

No	Pathological results	Treatment	Radiotherapy technique	Margin	Recurrence	Follow-up (months)
1	NPAC	S + R_post	IMRT	R1	No	78.6
2	NPAC	S + R_post	IMRT	Close	No	73.2
3	NPAC	S	None	R0	No	35.9
4	NPAC	S + R_post	IMRT	Close	No	80.9
5	ACC	S	None	R0	No	39.4
6	ACC	S + R_post	IMRT	R1	No	78.9
7	Clear cell carcinoma	S	None	Indeterminate	No	30.7
8	NPAC	S + R_post	IMRT	Close	No	67.0
9	ACC	S + R_post	IMRT	Indeterminate	No	63.1
10	MEC	S + R_post	IMRT	R0	No	74.1
11	ACC	S + R_post	IMRT	R0	No	5.4
12	ACC	S + R_post	IMRT	R0	No	12.0
13	NPAC	S + R_post	IMRT	Indeterminate	No	24.0
14	NPAC	S + R_post	IMRT	Indeterminate	No	4.5
15	NPAC	S + R_post	IMRT	Close	No	24.0
16	NPAC	S	None	R0	No	9.8
17	NPAC	S + R_post	IMRT	Indeterminate	No	72.0
18	ACC	S + R_post	IMRT	Indeterminate	No	24.0
19	NPAC	S	None	R0	No	84.0
20	NPAC	S	None	R0	No	60.0

ACC = adenoid cystic carcinoma, IMRT = intensity-modulated radiation therapy, NPAC = primary nasopharyngeal papillary adenocarcinoma, R-post = postoperative radiotherapy, S = surgery.

### 3.3. Treatment-related complications

Postoperative complications were generally mild and manageable. Minor bleeding occurred in one patient (5%), and no cerebrospinal fluid leaks were observed. Dermatitis was the most common acute toxicity, occurring in all patients who received radiotherapy (100%). Late complications were uncommon, including olfactory dysfunction in 5 patients (25%) and chronic rhinosinusitis in 2 patients (10%) (Table 3).

**Table 3:** Treatment complications.

Characteristics	Number of patients (n/N)	%
Acute complication		
Minor bleeding/ epistaxis	1/20	5
Cerebrospinal fluid leak	0/20	0
Dermatitis	14/14	100
Late complication		
Olfactory dysfunction	5/20	25
Chronic rhinosinusitis	2/20	10

## 4. Discussion

**Figure 1.**
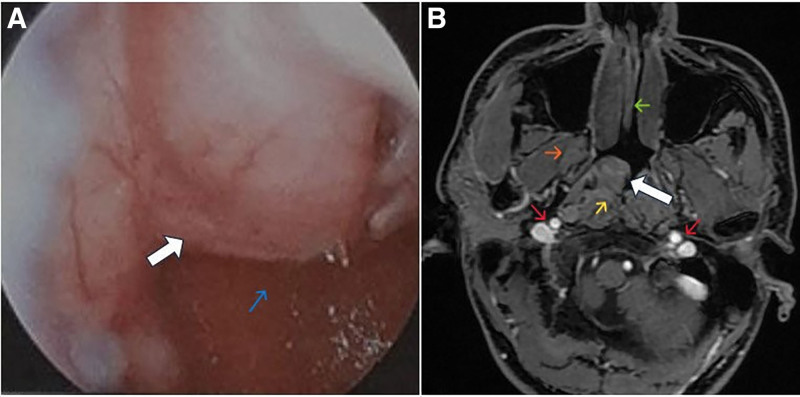
Nasopharyngoscopy (A) and contrast-enhanced axial MRI (B). Large white arrow: nasopharyngeal mass arising from the roof of the nasopharynx with right-sided predominance. (A) Blue arrow: posterior nasopharyngeal wall. (B) Green arrow: nasal septum; red arrows: internal carotid artery and internal jugular vein; orange arrow: medial pterygoid muscle; yellow arrow: nasopharyngeal roof.

A total of 20 patients with pathologically confirmed nasopharyngeal papillary adenocarcinoma or salivary gland–type nasopharyngeal carcinoma were included in this study, with a median age of 50.5 years (range, 16–64) and a male-to-female ratio of 6:14. Earlier WHO classifications grouped nasopharyngeal tumors arising from mucous epithelium or glandular tissue under the umbrella term of adenocarcinoma, including both NPAC and salivary gland–type carcinoma.^[5]^ The current WHO classification, however, recognizes nasopharyngeal adenocarcinomas and salivary gland–type carcinomas as distinct entities.^[1]^ These tumors are rare, accounting for approximately 0.38 to 0.48% of all nasopharyngeal malignancies.^[2]^ Nasopharyngeal papillary adenocarcinoma was first described in 1988 by Wenig et al.^[3]^ Patients range in age from 7 to 77 years, with a female predominance.^[6]^ In our NPAC cohort, the median age was 48.0 years, and female patients accounted for the majority of cases (66.7%). In comparison, population-based studies of salivary gland–type nasopharyngeal carcinoma have reported a higher median age at diagnosis, typically in the sixth to eighth decades of life. Local symptoms of nasopharyngeal tumors mainly depend on the extent of the primary tumor. In our study, the most common presenting symptoms were nasal bleeding (40%) and nasal obstruction or congestion (25%), followed by tinnitus (15%) and headache (5%). Notably, 15% of patients were asymptomatic and were diagnosed incidentally during routine examination. Currently, the etiology for these tumors remains unclear. In our study, EBV testing was performed on peripheral blood samples using quantitative PCR to measure plasma EBV-DNA levels. Although a majority of nasopharyngeal cancers are closely related to Epstein-Barr virus infection, the same has not yet been established for NPACs and SGNPC. This observation is consistent with our findings, as all tested patients in our cohort were EBV-negative, though missing EBV testing in some patients may limit interpretation regarding EBV association in this cohort. The previous reports have shown that nasopharyngeal papillary adenocarcinoma was commonly exophytic with a papillary, nodular or polypoid appearance and most frequently in the posterior or roof of the nasopharyngeal walls.^[6]^ In our study, regarding tumor location, all NPACs were located at the roof of the nasopharynx, accounting for 100% of cases, whereas SGNPCs showed a distinct distribution, with the majority arising from the lateral wall of the nasopharynx (75.0%). This difference suggests a divergent anatomical predilection between the 2 pathological subtypes. Figure 1 illustrates a representative case of NPAC. Nasopharyngoscopy showed a polypoid lesion on the roof of the nasopharynx, and MRI revealed a 28 mm tumor at the same location with extension into the parapharyngeal space. Low-grade NPAC is characterized by papillary structures with hyalinized fibrovascular cores lined by cuboidal to columnar cells, which share morphological similarities with papillary thyroid carcinoma. One of the most prominent features of NPAC is positive immunohistochemical reactivity for TTF-1. However, TTF-1 is not specific to NPAC and can also be expressed in papillary thyroid carcinoma. Thyroglobulin is useful in differentiating NPAC from metastatic papillary thyroid carcinoma, as it is typically positive in thyroid carcinoma but negative in NPAC. Other markers reported in the literature, including PAX8, CK19, and HBME-1 are frequently positive in papillary thyroid carcinoma and may aid in reinforcing that diagnosis. In contrast, primary nasopharyngeal papillary adenocarcinoma typically lacks PAX8 and thyroglobulin expression.^[7]^ In addition, positive immunohistochemical staining for CK7, vimentin, and EMA in some cases may further support the diagnosis.^[8]^ In our cohort, most NPAC cases were diagnosed based on hematoxylin and eosin (H&E) morphology. Immunohistochemical studies were performed in 6 of 12 cases for differential diagnosis with metastatic papillary thyroid carcinoma. All 6 tested cases were negative for thyroglobulin and positive for both TTF-1 and EMA. We used the 8th edition of the AJCC for staging. Our findings regarding NPACs are consistent with previous studies, showing that the tumors are usually confined to the nasopharynx without lymph node involvement. Rarely, as reported by Dewi Susilowati, the tumors can invade the medial wall of the orbital cavity and present with bilateral lymph node metastases.^[9]^ However, whether SGNPC typically presents at an early or advanced stage remains controversial. Some studies have reported that more than half of patients present with advanced-stage disease (stage III/IV).^[2,10]^ In contrast, most patients in our cohort presented with early-stage disease T1-2 (90%), and no cervical lymph node metastasis was observed. This finding may be partly attributable to selection bias, as patients with advanced disease were less likely to undergo definitive surgical treatment at our institution. The most common histological subtype of SGNPC was adenoid cystic carcinoma (6 cases, 75%), followed by mucoepidermoid carcinoma (1 case, 12.5%) and clear cell carcinoma (1 case, 12.5%). Although results varied among studies, most reported that adenoid cystic carcinoma was the one of the most common histologic type.^[11,12]^ The same finding was reported in previous studies of primary salivary gland–type carcinoma of the nasopharynx, where metastatic lymph nodes mainly occurred in adenocarcinoma, and no patients with ACC or mucoepidermoid carcinoma were found to have metastatic cervical lymph nodes.^[10]^

With a median follow-up of 49.7 months, all patients in this study were alive at the last follow-up, and no locoregional recurrence or distant metastasis was observed. In our cohort, papillary adenocarcinoma was associated with good outcomes. However, no formal statistical comparison could be performed due to the absence of events, and these findings should be interpreted with caution as they are hypothesis-generating in this small, surgically selected cohort. Unlike conventional nasopharyngeal carcinoma, NPAC is an indolent tumor characterized by a polypoid appearance and slow growth rate, for which surgical excision appears to be the most appropriate treatment. The benefit of adjuvant radiotherapy following complete resection of early-stage NPAC remains uncertain. Several studies have suggested that adding PORT does not improve survival in patients with negative margins and no adverse prognostic features, such as lymphovascular or perineural invasion, while potentially increasing treatment-related toxicity.^[13]^ In the NPAC cohort (n = 12), margin status was R0 in 4 cases, R1 in 1 case, close (<2 mm) in 4 cases, and indeterminate in 3 cases. Currently, no international consensus guidelines clearly define the indications for PORT in early-stage NPAC; however, surgical resection remains the primary treatment modality, and margin status is an important factor in guiding adjuvant therapy decisions. For patients with positive (R1/R2) or close margins, PORT is generally considered beneficial based on broader evidence from head and neck oncology. In the 3 indeterminate cases, treatment decisions were made after multidisciplinary discussion with the surgeon and careful review of pre- and postoperative imaging, leading to the recommendation for adjuvant radiotherapy. At the end of the study, no locoregional recurrence or distant metastasis had been observed in any margin subgroup. However, longer follow-up is required to better evaluate oncologic outcomes and to meaningfully compare results across different margin status strata. All patients with SGNPC were alive, with no evidence of local recurrence or distant metastasis. The favorable outcomes observed in this cohort may be related to early-stage presentation and complete surgical resection; however, selection bias inherent to the retrospective design and surgical case selection should be considered when interpreting these results. Due to the relatively low sensitivity to radiotherapy, surgery may represent the preferred treatment for selected patients with limited-stage SGNPC (T1–T2). In patients with well-differentiated tumors, DFS and OS were significantly better in the surgical treatment group compared to the non-surgical group.^[10]^ Minor salivary gland-type tumors of the nasopharynx are associated with poorer outcomes when compared to papillary adenocarcinoma and the other minor salivary gland tumors of head and neck sites.^[2]^ Findings from literature have shown that advanced-stage tumors (stage III/stage IV) and advanced age (≥80 years) were associated with worse outcomes.^[14]^ Salivary gland histologic subtype and tumor grade are significant factors for prognosis. Similarly, recent studies have demonstrated that lymph node metastasis is significantly correlated with overall survival in patients with salivary gland-type malignancies of the head and neck region.^[15–17]^ An analysis of salivary gland-type cancer of nasopharynx in US indicated that Mucoepidermoid carcinoma demonstrated the best survival outcomes.^[2]^ According to a recent study, tumor histology strongly affects survival: the 5-year survival rate was 79% for low-grade tumors versus 31% for high-grade tumors.^[18]^ Radiotherapy plays an important role in specific clinical scenarios: as postoperative therapy for advanced-stage disease (T3/T4 or node-positive tumors) or in patients with high-risk pathological features (high-grade histology or adenoid cystic carcinoma); as primary treatment, with or without chemotherapy, for inoperable cases; and with a limited role in the preoperative setting for selected patients.^[19]^ Postoperative complications were generally mild and no severe acute or late toxicities were observed. Dermatitis occurred in all patients receiving radiotherapy, while late complications such as olfactory dysfunction (5/20) and chronic rhinosinusitis (2/20) were uncommon. These results indicate that endoscopic endonasal surgery, with or without adjuvant radiotherapy, is a safe and feasible treatment for NPAC and SGNPC.

This study has several limitations and potential sources of bias. Firstly, it employed a retrospective, observational design, relying on the completeness and accuracy of medical records. Additionally, given that SGNPC as well as NPAC are rare diseases, the sample size was limited. Consequently, the findings should be interpreted with caution, and no definitive clinical recommendations can be made based on this study alone.

## 5. Conclusions

Primary nasopharyngeal adenocarcinoma and salivary gland-type carcinoma are rare malignancies. In our cohort, the majority of patients presented at an early stage, and surgical resection was the primary treatment modality with favorable outcomes. However, these findings should be interpreted with caution given the small sample size and the surgically selected, early-stage nature of this cohort, and should be considered hypothesis-generating rather than definitive. Further prospective studies with larger patient populations and longer follow-up are needed to better define optimal treatment strategies and long-term outcomes for these rare tumors.

## Acknowledgments

The authors declare that they have no acknowledgements to report.

## Author contributions

**Conceptualization:** Dang Nguyen Van, Nha Hoang Van, Toan Tran Trung.

**Data curation:** Dang Nguyen Van, Duc Nguyen Dinh, Toan Tran Trung.

**Formal analysis:** Duc Nguyen Dinh, Nha Hoang Van.

**Methodology:** Dang Nguyen Van, Duc Nguyen Dinh, Nha Hoang Van.

**Software:** Duc Nguyen Dinh.

**Supervision:** Dang Nguyen Van.

**Validation:** Dang Nguyen Van.

**Visualization:** Toan Tran Trung.

**Writing – original draft:** Dang Nguyen Van, Duc Nguyen Dinh.

**Writing – review & editing:** Dang Nguyen Van, Duc Nguyen Dinh.
